# Japanese International Medical Graduates and the United States clinical training experience: Challenges abroad and methods to overcome them

**DOI:** 10.1002/jgf2.315

**Published:** 2020-04-29

**Authors:** Brian S. Heist, Haruka Matsubara Torok

**Affiliations:** ^1^ Department of Medicine University of Pittsburgh School of Medicine Pittsburgh Pennsylvania; ^2^ Department of Medicine University of Minnesota Medical School Minneapolis Minnesota

**Keywords:** medical education, medical migration, postgraduate medical education, qualitative research

## Abstract

**Introduction:**

Due to the large language and cultural distances between Japan and the US compared to many countries, Japanese International Medical Graduates (IMGs) may have a different US training experience, including more stress, than many IMGs. We examined the US clinical training experience for Japanese IMGs, including the challenges encountered, how those challenges are overcome, and the benefits of US training.

**Methods:**

We performed individual semistructured interviews with 35 purposively sampled Japanese IMGs who had completed US clinical training. Exploratory thematic analysis was conducted using iterative data collection and constant comparison.

**Results:**

All participants reported high personal growth and that US clinical training was worth the sacrifices. Commonly fatigue was lower than during Japanese residency. Participants explained medical practice and local culture associated challenges that aligned with literature on US graduates and other IMGs. By contrast, nearly all participants reported that English communication was very challenging, and described specific language related struggles and methods to help overcome them. Communication struggles were contextualized within an American training culture that values verbal assertiveness. Self‐esteem varied among participants and, for some participants, improved with confidence in communication. Several participants reported depression and other mental illness. The training environment varied among residency programs.

**Conclusions:**

Japanese IMGs who completed US training report that it was worth it, but describe significant language and culture related struggles and effects on mental health. Further research should address which Japanese IMGs are most likely to struggle, how this will transpire, and how to optimize the US clinical training experience.

## Abstract 2

Individual interviews were conducted with thirty‐five Japanese international medical graduates who had completed US clinical training, in order to understand the challenges during US clinical training and methods used to overcome them, and the self‐esteem, fatigue, and personal growth experienced. Main themes include the consensus that US clinical training was worth the sacrifices, but that English communication was tremendously challenging and exacerbated by an American training culture that values verbal assertiveness. Commonly, Japanese IMGs attempted to compensate by striving to perfect other components to their performance, such as medical knowledge and professionalism. Several participants reported depression and other mental illness.

## 1 INTRODUCTION

To some extent, in their pursuit of United States (US) clinical training, all international medical graduates (IMGs) share personal sacrifices including time and expenses for the US Medical Licensing Examinations and application process, and the opportunity costs of other activities and personal relationships.^1^, ^2^ Within the literature, the elaborated portrait of this endeavor explains IMG anticipation of improved salary, career opportunities, and social conditions, intertwined with cultures of migration within their home medical institutions.^2^, ^3^, ^4^, ^5^, ^6^ However, the literature, while frequently examining IMG ethnic subsets individually, is shaped by the predominance of IMGs hailing from lower income nations and a medical school education conducted in English.^3^ By contrast, Japanese IMGs are distinguished from most IMGs by a greater challenge to achieve English proficiency and by the primary incentives of a personal challenge to become a more accomplished doctor and desire to improve medical education back in Japan.^7^

Literature examining the IMG experience during clinical training in the host country commonly addresses IMGs of different ethnic backgrounds collectively and identifies struggles frequently with the unfamiliar healthcare system, regional accents and vernacular, and incongruences with local values and their own backgrounds.^8^, ^9^, ^10^, ^11^ At the same time, the literature finds that compared to fellow American resident physicians, IMGs experience lower fatigue, higher self‐esteem, and higher personal growth.^12^ Within these studies, Japanese IMGs are not represented.

In consideration of the steeper English language barrier and nuanced cultural differences,^13^, ^14^ the Japanese trainee relationship to the US experience may not match common IMG themes. Potentially relevant aspects of American graduate medical education (GME) contrasting Japan include strict duty hours, and regimented progressive responsibility and conditional independence intertwined with written examinations and evaluations during and upon completion of training.^15^ The underlying objectives are to assure safety for all patients and competence of all physicians.^16^ An important consequence is that the American system mandates remediation programs for trainees who demonstrate unsatisfactory performance, and ultimately dismissal of trainees who fail to satisfactorily improve.^15^ Attrition rates during residency are higher among IMGs than US graduates.^17^ Such practice contrasts Japan where long‐term employment is generally assured upon entrance to a training institution; in turn, for Japanese IMGs, US clinical training may incur significant stress.

Individual Japanese IMGs have written about their US clinical training experiences,^18^ but a systematic study is lacking. In this study, we examine the US clinical training experience for Japanese IMGs, including the challenges encountered and where possible, how those challenges are overcome, and whether the US training was worth the sacrifices made. We hope to assist interested Japanese and enhance understanding of international medical migration through the lens of this atypical population.

## 2 METHODS

This report is complemented by a separate report from the same study that examines challenges for Japanese physicians in their pursuit of US clinical training.^19^ We used identical participants and methods, and collected and analyzed the data simultaneously.

### 2.1 Clinical training context—the American Graduate Medical Education system

In the United States, the American College of Graduate Medical Education (ACGME) oversees GME for nearly all clinical specialties, including the duration and content of training, with input from specialty professional societies.^20^ Entry into medical care for most patients and clinical scenarios is via primary care,^21^ and accordingly, the primary care fields of internal medicine, family medicine, and pediatrics are allotted the most residency positions.^22^ Similar to the evolving Japanese clinical training structure,^23^ a three‐year training program in these fields must generally be completed prior to subspecialty fellowship which lasts an additional 1‐3 years. A comparable organization exists in surgical fields whereby general surgery residency is a foundational step to specialized fellowship in some disciplines. Completion of each residency or fellowship training program is followed by a rigorous board certification examination.^20^ Board certification is increasingly required by physician employers and insurance companies.^24^

### 2.2 Study approach

Our study employed constructivist exploratory thematic analysis,^25^, ^26^ given our intention to understand the US clinical training experience for Japanese IMGs. Constructivist methodology recognizes the relationship of researchers' prior knowledge to data collection and interpretation.

### 2.3 Participant sampling

Because no comprehensive database of Japanese IMGs was available, potential participants were identified by (a) asking participants to suggest other potential participants, optimally with experiences contrasting their own, and (b) requesting names of Japanese graduates from training programs in the United States and Japan who had educated Japanese IMGs. Graduation from Japanese medical schools and completion of US clinical training within one to fifteen years at time of interview were required for participation. Also, we targeted approximately 50% returnees to Japan and 50% practitioners in the United States. We selected these parameters because separate work examines experiences after US training. Each participant was solicited via email with a description of the study and provided informed consent. Thirty‐five of 39 contacted physicians agreed to participate. The Institutional Review Board at the University of Pittsburgh Medical Center approved the study.

### 2.4 Data collection and analysis

We conducted individual semi‐structured interviews from February 2013 until October 2015 in person when feasible, and otherwise via phone or Skype. Interviews were conducted in English, digitally recorded, and transcribed verbatim by a professional transcriptionist.

We used open coding methodology to independently analyze the transcripts, and through discussion created an initial codebook. Interviews and transcript coding then progressed in an iterative fashion; through constant comparison of codes from current interviews with codes from prior interviews, we modified questions and probing techniques in subsequent interviews. We discussed the emerging themes and resolved any disagreements through discussion. The software Atlas.ti 7.0 (Scientific Software) facilitated the coding process. Further methodology details are explained in the accompanying work.^19^

In this study, participants were asked about (a) challenges during US clinical training with follow‐up probing questions where applicable that included influences and context, and methods to overcome the challenges; (b) their fatigue, self‐esteem, and personal growth during US training compared to during Japanese training to house this study within the literature on IMG experiences in the US;^12^ and (c) the biggest benefit of pursuing clinical training in the United States. As a validity check, two participants confirmed the emerging themes.

To improve readability of quotations, we edited the grammar and, where thoughts were not verbalized, added bracketed text.

## 3 RESULTS

### 3.1 Participant characteristics

Of the 35 participants, thirty‐four completed US residency in 8 disciplines at 8 different US institutions. Thirty‐one participants completed residency in the primary care fields of internal medicine, family practice, or pediatrics (American GME includes residency programs combining clinical disciplines and accommodates completion of residency in multiple clinical disciplines). Remaining residencies represented were general surgery, anesthesiology, emergency medicine, neurology, and pathology. Twenty‐four of 35 participants completed fellowships in 14 disciplines at 17 different US institutions. Twenty‐one of the 31 participants who completed residency in primary care fields also completed fellowship training in one or more of the following fields: infectious disease, geriatrics, hematology/oncology, pulmonary/critical care medicine, rheumatology, cardiology, gastroenterology, public health, preventive medicine and occupational health, faculty development, clinician scientist training, and medical education. Remaining fellowships represented were transplant surgery and thoracic surgery. One participant entered directly into clinical fellowship. Additional participant characteristics are reported in the accompanying study.^19^

For the purpose of quotations, participants are numbered 1‐35. At the time of the interview, participants 1‐19 and 20‐35 were working in Japan and the United States, respectively.

### 3.2 Challenges during US clinical training

#### 3.2.1 English communication

Foremost, nearly all participants reported that English was a major challenge. Communicating at the pace of natural conversation on diverse topics and sometimes with strong accents and/or local vernacular was significantly different from participants' prior experiences in relatively controlled scenarios. As Participant 8 succinctly explains, “[Upon beginning US residency] I thought I knew some English language, but I did not.” Challenges included communication, in general and in specific situations (Table 1). The language barrier could be particularly stressful for participants with extensive clinical experience in Japan.

I was already an attending [in Japan,] and then after starting US residency, I wasn't [included] in discussions, especially [during] critical patient care situations, because of the language barrier. That gap was striking. (16)

**Table 1:** English challenges during US residency

Challenge	Representative quotes
English communication in general	As a clinician, I didn't feel behind compared to other [residents.] But [when it came to] understanding discussions, I always had difficulty. When I talked with patients, if I spoke slowly they would speak slower,… but if they started talking with their family, I could not follow them… [Similarly] when my attending and a consultant were talking to each other, I sometimes could not follow the discussion… I felt my ears never improved. (7) I would catch 70%‐80% [of the conversation], so that's quite a disadvantage… I felt like my IQ was down to 30 points or something. (28) I didn't have much difficulty with listening, but speaking was a challenge… so I wrote down and memorized every single phrase used in clinic. (25) I had more experience than American residents… but I couldn't explain, I couldn't persuade, so sometimes it was difficult. (22) I thought my English was much better [than it was]. I didn't understand subtle nuances, so it was difficult for me to figure why a patient was refusing some procedure or medication. (9)
Dictation	[When dictating,] I think most Japanese IMGs, at first, actually wrote a draft and then read it [into the phone], which doesn't make sense, but we were very afraid of making mistakes. (11)
Communication with American colleagues	When I was presenting over the telephone, I could feel the other doctor becoming frustrated with my English… If I [needed] a long pause to find the right word, sometimes residents who [did not know me well] would say, “never mind,” and carry on without listening to my whole presentation. (25)
Non‐native English‐speaking patients	In [the city where I trained], there are so many people who do not even speak English. If the patients do not speak English, that means that both of us are speaking a second language. It was so hard to get a sufficient history and I was not able to understand their social or cultural background enough‐ it was very tough. (8)
Communication with patients	In Japan, the English we are exposed to is very easy to understand, but in the States, the patients speak with a variety of accents… That was hard. (3) I still don't understand some colloquialisms… But patients understand I’m a foreigner, so I always try to ask exactly what they mean, by, [asking] say, A, B, or C. (24)
Emotionally delicate situations	The patient was a 15 year‐old girl, and her mother was in the room. The patient was sexually active, and it seemed like she wanted to discuss it with me. I asked the mother to step out of the room, and she got mad. She actually said, “OK, I’ll leave.” But about ten minutes later, she returned, yelling at me “Why did you make me step out of the room?! I’m her mother! I have a right to know everything about my daughter!” And I was like, “That was my fault that I said that, but she has the right to talk to me individually, and you have to leave the room.” And she left the room but later asked to talk to the director. I learned through [role playing with a] standardized patient, that the way I spoke was rude. (3)
Nonmedical situations	Because I had completed internship at a US Naval Hospital [in Japan], US residency was relatively easy for me. Without it, it would have been much more difficult. I didn't have much trouble with medical English or the medical care. My difficulty was living in [a foreign culture,] such as finding an apartment, finding a car, getting it repaired when it was broken, etc (17)
Public speaking	My English was poor and I was not used to presenting in public, so I was very nervous. I needed to practice a lot. Even at the end of residency, I did not feel good about my presentations. (10)

Participants did not perceive discrimination from coworkers or patients for being Japanese but did report infrequent tension during interactions that they attributed to their language barrier.

Overall, people were really supportive of Japanese physicians. Sometimes Americans had [a negative impression due to my] English communication skills, and the really strong accent I had. (13)

#### 3.2.2 American medical practice and local culture

Participants described cultural differences from Japan (Table 2). Some issues were specific to medical practice. Many participants reported that a strictly enforced shift schedule with frequent sign‐outs resulted in initial difficulty completing patient care tasks including documentation more detailed than in Japan, and internal ethical struggles when they were unable to be nearby for patients such as during terminal illness. Relatedly, participants explained that the American model of care prioritizes discharge sooner than in Japan and that collaboration among physicians representing numerous disciplines combined with extensive ancillary support accelerates patient management. Patient care decision making was rapid, and communication among healthcare professionals was more extensive than in Japan. Participants noted that decision making was complicated by the American multi‐payer health insurance system, whereby financial barriers to care may arise depending on the patient's insurance provider and policy. Other challenges—ethical stances in certain situations, the doctor‐patient relationship, excessive opioid analgesics, and patients representing diverse ethnicities and socioeconomic extremes—were more intertwined with American national cultural differences from Japan. Additionally, a few participants lamented that the American diet was less palatable than back home.

**Table 2:** Medical practice and local culture‐associated challenges during US residency

Challenge	Representative quotes
Duty hours and goals of training	In Japan, I think it's considered a good thing to stay in the hospital, at the patient's bedside, as much as possible. You are not necessarily obtaining new knowledge or skills… But in the United States, it's completely different. It's better to be an efficient learner. You should finish your stuff within the timeframe that you're allowed… You cannot be there over 80 hours per week. (15) My patient was dying and at 8 or 9 PM, I was staying in the hospital and then my [senior] resident said, “Why don't you go home?” [I wondered,] “Can I go home?” I was really worried about my patient through the night. The next morning, I came in at 6 o'clock and talked to the night float resident who just replied, “Oh, he died.” I felt so bad that I was not with the patient at the time. It took me a while to understand that's the cultural and professional norm for physicians in the States. (24)
Documentation expectations	Clinical reasoning was a big issue for me. I really needed the mindset to write everything that I’m thinking. It was a challenge. (1)
Quick decision‐making pace	In Japan, patients [commonly] are hospitalized for five to seven days, without doing anything, so we have time to think before making [management] decisions. [But in American hospitals] we must make decisions really quickly; we admit patients overnight and then discharge them really fast. (27)
Intense collaborative care model	I was so shocked by the many phone calls that I had to make‐ to social workers, to consultants, signouts. I didn't know how to call consults. [There is] so much communication [for patient care compared to in Japan.] (34)
US health insurance system	I didn't know anything about health insurance in America when I started residency, so that was really hard. For example, when I ordered an MRI but it was not approved by Medicare, sometimes I had to do a lot of paperwork. The system was hard [to navigate] even when a social worker helped. (3)
Medical ethics	In the case of code status, in Japan we try not to intubate old people because we cannot extubate them based on Japanese law. I was shocked [by US practice,] because many people were intubated and then extubated very easily. That kind of culture difference was a little difficult to adjust to. (14)
Doctor‐patient relationship	In the United States, [patients often] come seeking, and demanding, and I think they want to have more of a constructive discussion with the physician, because they are also responsible for their health, right? (23) Patients in the United States are more blunt, meaning that they can criticize your conduct. (31) I think there are many [more] noncompliant patients compared to in Japan. (34)
Opioid prevalence	[In Japan, some] patients don't notice that they are dependent, but keep asking for benzodiazepines. American patients tend to ask for narcotics and some patients kept asking. That was really stressful. (3)
Patient ethnic and socioeconomic diversity	I was at a [religiously affiliated] institution. You need to make conversation. If you don't know anything [about the other's] cultural or religious background, it's very difficult. (29) [At my program,] there were many patients and families who just don't have enough social support. These patients have a tough life. [And sometimes] they have cultural and language barriers… I have worked in Japan with relatively underserved people, but they didn't have this amount of difficulty. (30)
Daily living	I thought I would like hamburgers, but actually I only ate hamburgers 3‐4 times during the whole 3 years. I think the calories may be too high [in American meals], but you still have to eat it all. I was gaining more and more weight, but I was not really satisfied with the quality of the food. (14) I really missed Japanese food. It was at [a midwestern program.] The level of food was not that good. I did not have any good ramen or sushi at all. (19)

#### 3.2.3 American clinical training culture and the training program environment

Intertwined with the challenge of English communication and differences in medical practice and local culture, participants described the challenges of verbal assertiveness and expressing one's opinions. These skills influenced perceived competence and were important especially during negotiations, for example for scheduling work shifts, and during the continuously occurring clinical discussions. The backgrounds and personalities of other trainees affected the significance of these issues and the stress of the training experience. Table 3 contains representative quotes.

**Table 3:** Training culture‐associated challenges during US residency

Challenge	Representative quotes
Assertiveness	You must speak up and say that you learned something, otherwise they will say you're not doing anything, or you're not thinking. So you actually have to tell them what you are thinking and what you have done to let them trust you. (15) In the US you have to speak up. “I want this, I don't want this.” Otherwise you may receive extra work. I didn't know that… For the first one or two years, I think the duration and number of call assignments, etc was unfair. (19)
Expressing one's opinion	In Japan, we are not really trained to discuss. [In the US] I had to express my opinion [which was challenging.] … Asians, not Indians, but other [East] Asian IMGs, I think have difficulty. Just like me, they can't speak up. They can never be dominant in the group. (32) During attending teaching rounds… even if I know the topic, if I cannot communicate my knowledge well, I will be left out of the discussion and [my reputation will be affected.] (5)
Competition among residents	Most of the residents were from South Asia and Eastern Europe. It was a very competitive atmosphere… There was a lot of political jockeying. (26)

The presence of other IMGs in a residency program was consistently favorable to nonexistence, but native English‐speaking IMGs were distinguished from others given the significance of language to the training experience. The most comfortable programs were those with prior, and ideally, other current Japanese IMGs.

[Preceding] Japanese residents worked so hard and created a great reputation [for Japanese physicians] in the hospital. (24)

The program did not understand what Japanese residents were suffering from. But they understood upper level Japanese residents know what we need to survive. Therefore, they assign struggling Japanese residents to work with Japanese senior residents… [Fortunately, the program had] many prior Japanese IMGs. (18)

In my program there were many foreigners [including Japanese] during residency. That made the work environment more comfortable. If almost everybody was US graduates, probably it would be more stressful. (8)

My program had other IMGs, [but they were] from India and Canada, and one from Australia; they were native English speakers. (7)

My US residency program was not used to IMGs. [In general] the staff tried to help me… [At the same time, the region] has a physician shortage problem so they prioritize residents from there. Sometimes I felt unwelcomed, because I was from Japan and planned maybe to return after training. (15)

#### 3.2.4 Potential for dismissal from training program

Several participants explained the pressure felt from the awareness that sometimes US clinical training programs dismiss trainees, including Japanese IMGs.

In Japan, you're basically guaranteed to have your position, but in US training if your [In‐Training] Exam score is poor and your [daily] performance is not very good, your position [is not guaranteed.] Knowing it was possible for me to be kicked out was stressful. (4)

#### 3.2.5 Mental illness

Several participants volunteered feeling depressed by the cultural and linguistic challenges and one participant reported a formal mental health diagnosis and treatment.

Some Attendings and classmates thought that I was really talented, but others got frustrated because that small difficulty with English slowed everything down and that was what they focused on… I was placed on remediation during which time I needed to work with four different Attendings. I was quite depressed that month…. (7)

Six months after starting residency, I developed insomnia, I couldn't decide, I couldn't put any orders in, so I feared that I couldn't continue training anymore and I better go back to Japan. I was introduced to a psychiatrist and starting counseling. [My diagnosis was] adjustment disorder…. I was placed on one‐month medical leave. (16)

No participants reported clinical incompetence as the source of such struggles.

### 3.3 Overcoming challenges during US residency

To cope with their primary stressor, English communication, participants most commonly described trying to compensate by enhancing aspects of work they could better control, such as medical knowledge and professionalism.

I was underappreciated because of my language. I felt like I had to [compensate by] working harder, [perfecting my] bedside manner and knowledge, [basically] everything. (19)

I had to work harder than US medical graduates, because I had to overcome that language barrier… My presentation skills may not have been good like an American native, so I had to make the content better, write my notes better, or see more cases. (23)

Sometimes I had a hard time expressing myself and I tried to compensate for this weakness by working and studying hard. Gradually other residents started recognizing me as a team member. During my 2nd year I scored high on the In‐Training Exam, and after that they looked at me differently. (2)

Additionally, some participants described specific methods they used to overcome English‐related challenges including an attitudinal shift toward accepting one's linguistic mistakes and asking for help, using nonverbal communication, using local students to help interpret, embracing one's identity as a trainee, and speech therapy. Table 4 contains representative quotes.

**Table 4:** Methods to overcome English‐related challenges during US clinical training

Method	Representative quotes
Not being afraid to make a mistake	I became not afraid of making mistakes when I spoke English. English is not my native language, so [making mistakes] is not shameful at all. After I [accepted] that, I could speak better actually. (17)
Asking patients to rephrase	On the phone many times I didn't understand the nurse or ER physician and [for months] would ask “Where are you?” Then I would go there and talk face‐to‐face. [Later] I learned and gained the confidence to ask the other person to rephrase what they said. (4)
Embracing nonverbal communication	I think because I can't say what I want to say, exactly, in English, it's almost like a technique [of mine] that I can sense what [patients are trying to communicate] by the way they act and talk, not just by their words. And then I can explain to them in a way they can understand. (32)
Spending time with patients	I tried to overcome [my difficulty in quickly building patient rapport] by seeing [hospitalized] patients more [frequently]. I sometimes felt that I’m getting credit every time. (6)
Using medical students	I couldn't follow what the patients were saying to me, due to their vernacular and accents. I used my medical students to help me out by having them interview the patients and then present to me. (13)
Identify yourself as a trainee	As a resident or fellow, I didn't have much stress, because patients are comfortable working with trainees…. Once I introduce myself, “I am a resident or fellow…,” patients know they have some attending backup and they do not really expect me to take responsibility. (8)
Speech therapy	The breakthrough for me was when as a second‐year resident in the States, I took a speech therapy course. [My program] actually paid almost $2000 for a speech therapist who provided one‐on‐one training… and fixed my accent. (27)
Extra English instruction	[My US training program] provided an English class and tutor. (33)

In response to the cultural differences, several participants explained the need for an adaptive attitude.

I didn't really compare Japan versus the US, I kind of felt “OK, this is the way it happens in the US” and then I just kind of got used to it. I don't think I struggled with those things. (21)

Similarly, some participants described the importance of adopting local practices.

I would steal styles from different people to make my own system. (32)

### 3.4 Fatigue, self‐esteem, and personal growth

Participants were surveyed about their levels of fatigue, self‐esteem, and personal growth during US clinical training. Most participants reported lower fatigue than during Japanese residency due to work‐hour limitations. Personal circumstances, such as parenthood, contributed to cases of increased fatigue. Participants evenly reported their self‐esteem in the United States as lower, similar, or higher than during Japanese training (Table 5). Factors lowering self‐esteem were the struggles with English, the foreign context, and regression to intern status described above. Participants with high self‐esteem tended to demonstrate confidence in English and feeling well‐prepared for US residency, for example in cases of prior US Naval Hospital internship. Some participants explained low self‐esteem during the first one to two years of struggles that rose with improved English communication, comfort with the American system, work performance recognition from colleagues, and associated confidence. All participants reported high personal growth during US training. Contributors included deepened understanding of a new culture, and professional and personal accomplishments.

**Table 5:** Fatigue and Self‐Esteem during US clinical training

	Number of Participants^a^		
	Lower	Similar	Higher
Fatigue during US residency compared to Japanese training	28	2	4
Self‐esteem during US residency compared to Japanese training	13	10	11

^a^ One participant did not complete any clinical training in Japan and is not included in these data.

### 3.5 Benefit of clinical training in the United States

All participants endorsed that in hindsight they would still pursue US training, but had varied opinions on the biggest benefit. Most commonly, they reported exposure to different medical and medical education systems.

You can compare [systems.] Maybe something that you experienced in [the other country] can help, at least locally. (20)

It definitely changes how you see things and how you think…. You become open‐minded… You know there's not only one answer to the question, so you learn to listen to other people. (15)

Numerous participants noted the exposure to the ethnic and racial diversity of the United States.

In the US, they accept diverse opinions… [and] people from other countries. (2)

Several participants identified US training as a bridge to a more global orientation.

When you pursue training in other countries, you come to understand what medicine really means in other countries. [Through that experience] you can become a global physician. I think the lack of that insight is a big limitation in Japan. (13)

Regarding specific content of US training, many participants emphasized the value of learning a national standardized approach to training and patient care; specific comments described learning evidence‐based medicine and developing skills in clinical reasoning, critically reading research studies, and performing clinical research. Several participants explained that work‐hour limits and rapid turnover of hospitalized patients taught them to work more efficiently. Participants in surgical fields cited more efficient accumulation of relevant case experiences. Several participants also described identifying mentors and role models in clinical fields underdeveloped in Japan, in research, and in administrative leadership.

[Dr X] showed me a great leadership model and she is still supporting the progress I am making here [in Japan.] I feel like I'm still supported here from people in the States. (1)

## 4 DISCUSSION

Japanese IMGs who completed US clinical training reported that the training was worthwhile for reasons most commonly linked to immersion in a different system and culture. They also described intertwined personal achievement contributing to high personal growth and lower fatigue than in Japan associated with American work‐hour limitations (WHL), which match the literature comparing US residency experiences for IMGs and US graduates.^12^

Japanese IMGs described some US clinical training challenges that align with those expressed by American physician trainees. These include the changing American infrastructure of medicine such as insurance payment models and abbreviated hospitalizations, changes to graduate medical education such as WHL, the opioid crisis, and America's extreme cultural and socioeconomic diversity where the most vulnerable populations are disproportionately cared for by GME facilities.^27^, ^28^, ^29^

Consistent with the literature on IMGs in US residency, Japanese IMGs denied that clinical knowledge and skills were a prominent challenge,^30^ but reported difficulty with local vernacular and accents, and sometimes the more collaborative Western doctor‐patient relationship.^10^ However, in contrast to themes describing the IMG experience,^12^ many Japanese IMGs described significant stress and sometimes low self‐esteem consistently attributed to communication struggles exacerbated by a foreign cultural context valuing verbal assertiveness. Associated devaluation of their performance pressured some Japanese IMGs to compensate by striving to perfect other elements of their work. Cases of depression and mental health diagnoses were reported.

Some Japanese IMGs blamed their communication struggles on poor listening comprehension and some emphasized difficulty explaining themselves. Others cited accent and nonverbal elements of communication. Effective communication is complex, requiring more than language proficiency. For example, literature on doctor‐patient communication identifies the importance of recognizing and appropriately addressing emotions, verbally and nonverbally, through vocal tone and body language.^31^ In their methods to overcome English‐related challenges, indeed several Japanese IMGs reported embracing nonverbal communication that was potentially enhanced by spending more time at the bedside.

Our findings suggest that certain factors might be protective against struggles in US residency, including an adaptive attitude, preceding internship at a US Naval Hospital in Japan, and US residency at a program with other non‐native English‐speaking IMGs—ideally prior and current Japanese IMG residents—located in regions with more Japanese immigrants. The stakes are high, as some participants mentioned Japanese IMGs who were dismissed from US residency programs. Our study design did not accommodate interviewing this population.

Our findings complement the literature on the IMG experience by providing insight into the challenges of a population facing steep linguistic and cultural differences in the host country, and, in some cases, methods to overcome such challenges. Measures to build on this research include examining the experiences of Japanese IMGs who commenced but did not complete US clinical training with the ultimate goal of predicting which candidates may struggle, how that may happen, and how to help them.

This study has limitations. Firstly, our study used qualitative methodology. In turn, the findings are hypothesis generating and might not be generalizable. Secondly, incomplete identification of themes may have resulted from our sampling strategy that relied heavily on participant referrals. Although the US training pursued by the participants overall aligns with fields traditionally underdeveloped in Japan,^32^, ^33^ experiences in some US clinical fields completed by Japanese IMGs may not have been represented. Thirdly, in the case of some participants, over a decade has passed since their US training. However, though Japanese medical training is undergoing intense reform,^23^, ^34^, ^35^ the predominant US training challenges to Japanese IMGs reported herein are linguistic and cultural and are not anticipated to have changed significantly in this interim.

## 5 CONCLUSIONS

Pursuit of US clinical training involves tremendous sacrifice, and Japanese IMGs who completed it report that it was worth it. Simultaneously, during US clinical training, these Japanese IMGs reported significant challenges and struggles. Some challenges reflect facets of American medical care, graduate medical education, and cultural and socioeconomic diversity that align with experiences of US graduates and other IMGs. Struggles more specific to Japanese IMGs were rooted in communication difficulties compounded by the Western culture valuing verbal assertiveness. Some Japanese IMGs experienced depression and other mental health diagnoses, and dismissal of Japanese IMGs from US training programs was mentioned. Topics for further examination include which Japanese IMGs are most likely to struggle, how this will transpire, and how to optimize the US clinical training experience.

## CONFLICT OF INTERESTS

The authors have stated explicitly that there are no conflicts of interest in connection with this article.
